# The Association of Endothelin-1 with Early and Long-Term Mortality in COVID-19

**DOI:** 10.3390/jpm13111558

**Published:** 2023-10-30

**Authors:** Lyudmila Turgunova, Irina Mekhantseva, Yelena Laryushina, Assel Alina, Irina Bacheva, Zhibek Zhumadilova, Anar Turmukhambetova

**Affiliations:** Department of Internal Medicine, Karaganda Medical University, Karaganda 100000, Kazakhstanbacheva@qmu.kz (I.B.); zhumadilova.z@qmu.kz (Z.Z.);

**Keywords:** COVID-19, mortality, endothelin-1, endothelial dysfunction

## Abstract

(1) Background: Endothelial dysfunction is a key mechanism in the pathogenesis of COVID-19. High endothelin-1 during COVID-19 is associated with severe complications and increased mortality rates during hospitalization. This study is aimed to investigate the association of endothelin-1 levels with the risk of 30-day and 12-month all-cause mortality in patients with prior COVID-19. (2) Methods: A prospective study was conducted involving patients with COVID-19 in Karaganda, Kazakhstan. The level of endothelin-1 in the blood serum was evaluated by ELISA. Univariate and multivariate Cox regression was used to determine factors and significance of endothelin-1 associated with the risk of mortality within 30 and 365 days from hospitalization. (3) Results: The median endothelin-1 was higher in the group of patients who passed away within 30 days. The group showed statistically significant differences when compared to healthy volunteers from the control group (*p* = 0.0001), surviving patients (*p* = 0.001), and those who passed away within a year (*p* = 0.002). (4) Conclusions: Endothelin-1 levels are associated with increased mortality risk during the acute period of COVID-19, while plasma endothelin-1 level association with COVID-19 survivor mortality risk does not persist after 12 months.

## 1. Introduction

The coronavirus pandemic has become an extraordinary public health challenge leading to extensive mortality worldwide. The WHO declared the end of the pandemic in May 2023, but more than 6.86 million people have died as a result of COVID-19 [1].

As the pandemic was gradually brought under control, the focus shifted to the long-term COVID-19 health effects on survivors. Initial studies showed that 30-day all-cause mortality and 30-day readmission rates for COVID-19 patients discharged for home oxygen therapy were 1.3% and 7.5%, respectively [2]. Moreover, 27% of patients were readmitted or died after discharge within 60 days [3]. Furthermore, patients after COVID-19 discharge within 6 months are also at risk of developing multiorgan dysfunction and a higher risk of death [4]. The cohort study of 13,638 patients demonstrated that the 12-month adjusted risk of all-cause mortality was significantly higher among patients with COVID-19 [5]. Uuskula et al. [6] confirmed that patients infected with SARS-CoV-2 had a three times higher risk of dying within 12 months compared to those not infected.

It is presumed that the post-COVID-19 manifestations may be determined to a greater extent by the severity of the initial episode of COVID-19 due to an increased level of inflammatory markers [7,8]. The mechanisms underlying the long-term effects of SARS-COV-2 remain to be determined. To date, growing evidence supports endothelial dysfunction as a key mechanism in the pathogenesis of COVID-19. Endothelial dysfunction in COVID-19 is associated with the degranulation of neutrophils, macrophages, and increased levels of cytokines [9,10,11,12,13]. On the other hand, it is assumed that SARS CoV-2 directly damages endothelial cells and causes systemic endothelitis with the development of multiple organ failure [14]. The endothelial dysfunction that develops in SARS CoV-2 is manifested by vascular imbalance toward vasoconstriction, followed by organ ischemia, inflammation, and a procoagulant state [15]. Autopsy investigations of COVID-19 deaths showed evidence of significant endotheliopathy [16].

Endothelial dysfunction is usually defined as decreased NO bioavailability and increased vasoconstrictor substances (such as endothelin-1 (ET-1), angiotensin II (Ang II), etc.) [17]. Endothelin is a vasoactive peptide consisting of 21 amino acids, represented by three isoforms, where ET-1 is the most widely expressed and therefore the most studied [18]. High concentrations of ET-1 are a marker for endothelial dysfunction of acute lung injury [19], primary pulmonary hypertension [20], and sepsis [21]. Recent reports suggest that higher levels of ET-1 during the initial stage of COVID-19 are associated with severe complications and increased mortality rates during hospitalization [22]. Willems et al. reported about the increased ET-1 levels in patients 3 months after their COVID-19 infection [23]. Presumably, endothelial dysfunction in the acute period with the development of several organ and system injuries can lead to unfavorable outcomes in the post-COVID-19 period. Moreover, there is an additional justification for considering the association of ET-1 with long-term mortality in patients who have undergone COVID-19. The ET-1 level was a powerful prognostic indicator of the development of 1-year mortality in patients with myocardial infarction [24], patients with pulmonary hypertension [25], and hemodialysis patients for 28-month supervision regardless of various clinical and biochemical variables [26]. Although the WHO declared the end of the COVID-19 pandemic, there is still a lack of data related to the association of ET-1 levels with the risk of acute and long-term mortality [22,27,28].

The aim of this study was to investigate the association of ET-1 levels with the risk of 30-day and 12-month all-cause mortality in patients with prior COVID-19.

## 2. Materials and Methods

### 2.1. Study Design and Participants

A prospective study was performed to enroll patients with COVID-19 who were hospitalized between May and August 2021 in the infectious diseases clinic of Karaganda regional clinical hospital and Karaganda Medical University Hospital. The inclusion criteria for the main group were patients over 18 years of age with confirmed COVID-19 who provided informed consent. COVID-19 was determined in accordance with WHO recommendations immediately upon admission to the hospital by RT-PCR taken from nasopharyngeal swabs or lower respiratory tract [29]. All patients with COVID-19 received glucocorticosteroids, antivirals, antibiotics, and anticoagulants according to the Kazakhstan COVID-19 treatment guidance [30]. Informed consent was obtained from either the patient or their accompanying person depending on the patient’s condition. Exclusion criteria: age under 18, pregnant and/or lactating women, immunocompromised patients (human immunodeficiency virus infection, active treatment for solid tumor and hematologic malignancies). The study included 470 participants, 291 (61.9%) women and 179 (38.1%) men. The average age was 59 years old, and the severity of their COVID-19 illness was assessed using WHO criteria [29]. The patients were divided into groups with moderate and severe severity depending on COVID-19 severity. The first group consisted of 382 patients with moderate disease severity, the second group included 67 patients with severe and 21 patients with critical disease severity. The control group of healthy volunteers was recruited through open sources and messages. The control group had to meet the following criteria: (1) a negative COVID-19 test result through RT-PCR, (2) no documented history of COVID-19, (3) no elevated COVID-19 antibody levels through ELISA, and (4) consent to participate by signing an informed consent form. The control group included 35 people: 23 (65.8%) women and 12 (34.2%) men. The average age was 48 years. This group was recruited as a reference group.

The study was approved by the Bioethics Committee of Karaganda Medical University No. 18, dated 14 April 2021. All analyzed data were evaluated as part of the clinical routine during hospitalization.

### 2.2. Data Collection

Upon admission, patients were thoroughly evaluated for complaints, socio-demographic data, comorbidities, previous drug therapy, and clinical manifestations. Additionally, their body mass index, heart rate (HR), oxygen saturation, and age-adjusted comorbidity index using the Charlson method were assessed [31]. The patients’ clinical data were evaluated during their hospitalization. Laboratory and instrumental data, intensive care unit (ICU) data, and cause of death were copied from electronic medical records. The percentage of lung tissue damage was assessed based on the results of computed tomography (CT) of the chest. The NLR index was calculated using the following formula: NLR = Absolute Neutrophil Count (ANC)/Absolute Lymphocyte Count (ALC). Laboratory and instrumental data, reasons for visiting a family doctor/specialist, and causes of death were obtained from electronic medical records during the year. Thus, patients were followed for a total of 365 days: 30 days after hospitalization to assess early mortality, then an additional 11 months to document long-term mortality for surviving patients. Patients who were still alive 365 days after hospitalization with COVID-19 were defined as survivors. The end point was all all-cause and cause-specific mortality, taking into account etiology and date of death.

### 2.3. Laboratory Analysis

Upon admission to the hospital or entry into the control group, blood samples were taken from individuals using venipuncture and collected in two vacuum tubes containing the anticoagulant EDTA, each with a capacity of 5 mL. Serum aliquots were then stored in a freezer at −80 °C. To measure ET-1 concentrations, an enzyme-linked immunosorbent assay (ELISA) was used with commercially available ELISA kits (Cloud Clone Corp., Wuhan, China) for ET-1 (#CEA482Hu), which has been analytically validated. The assay’s minimum detectable dose of ET-1 was less than 2.71 pg/mL, and its lower limit of detection (LLD) is determined as the lowest protein concentration that can be distinguished from zero by subtracting two standard deviations from the mean. The laboratory technicians who conducted the measurements were not aware of the patients’ characteristics or the specifics of the study.

### 2.4. Statistical Analysis

Statistical processing of the study data was performed using IBM SPSS Statistics for Windows, version 21 (IBM Corp., Armonk, NY, USA). Data were presented in graphs using GraphPad Prism 9 software (GraphPad Prism, San Diego, CA, USA). The normality of the distribution was assessed using the Kolmogorov–Smirnov test. Quantitative measures, given non-normal distribution, are described using median (Me) and interquartile range. Qualitative characteristics are described using percentages. For the non-parametric distribution of data, a comparative analysis of quantitative data between groups was carried out using the Mann–Whitney scale; for qualitative data, Pearson χ2 was used. Factors associated with the development of mortality within 30 and 365 days from hospitalization were analyzed by univariate and multivariate Cox regression. Hazard ratios were based on log-rank tests. Cut-off values for ET-1 were determined based on the median and used to assess differences in hospitalization survival using Kaplan–Meier survival analysis. Hazard ratios were based on the logarithmic test (Mantel–Cox). Statistical test differences were considered significant if *p* values were <0.05.

## 3. Results

The clinical characteristics of patients diagnosed with COVID-19 are presented in Table 1.

Over the entire observation period, a total of 35 patients died, with 20 of them (4.2%) dying within a 30-day period and an additional 15 people (3.2%) passing away in the following 11 months. The mean age of patients who died at one month and 12 months was higher than that of patients who survived (*р* = 0.0001 and *р* = 0.002). The number of concomitant cardiovascular diseases (presence of hypertension, history of myocardial infarction (MI), chronic heart failure (CHF)) was higher among patients with COVID-19 who died during the 30-day observation period compared to patients who survived. Patients with 30-day mortality had a higher percentage of lung damage (Me 45 (25–55), *p* = 0.005), higher respiratory rate (*p* = 0.0001), and lower oxygen saturation, compared with those who survived or had 12-month mortality. In addition, patients who died while hospitalized within a month were more likely to be transferred to the ICU (65%, *p* = 0.0001) and receive mechanical ventilation (60%, *p* = 0.0001).

It was observed that the early and late-death groups had higher neutrophil counts, NLR, and D-dimer levels. Furthermore, there was a significant difference in the white blood cell count (*p* = 0.01) and glucose level (*p* = 0.024) between patients who survived and those who died within the 12-month follow-up period.

**Figure 1:**
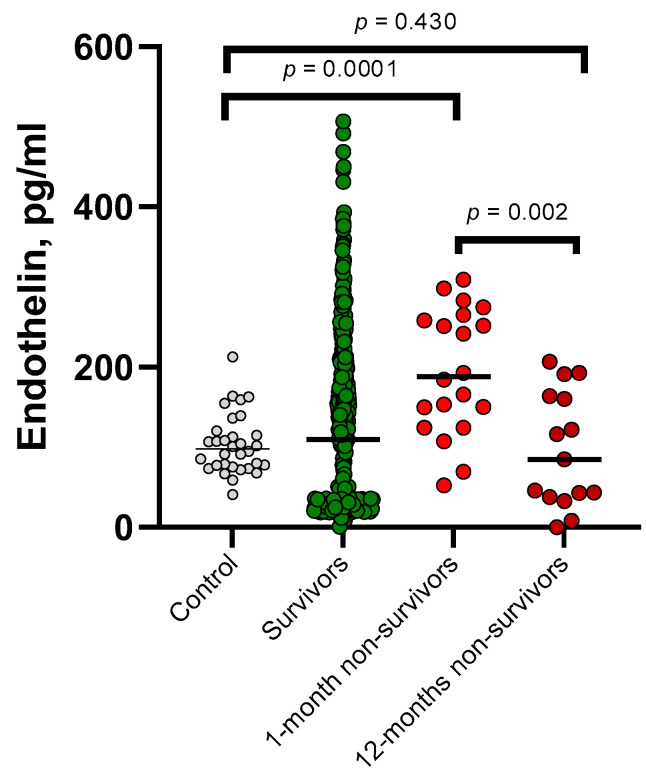
The plasma concentration of endothelin in survivors (*n* = 435), 1-month non-survivors (*n* = 20), 12-month non-survivors (*n* = 15), and healthy controls (*n* = 35) was analyzed and compared.

In a comparative analysis of ET-1 levels with early and long-term mortality (Figure 1). The median ET-1 was found to be higher in the group of patients who passed away within 30 days. This group showed statistically significant differences when compared to healthy volunteers from the control group (*p* = 0.0001), surviving patients (*p* = 0.001), and those who passed away within a year (*p* = 0.002).

Table 2 displays the outcomes of both univariate and multivariate Cox regression, which evaluate the correlation of markers with mortality after 1 month. ET-1 maintained a significant impact on the risk of patient mortality during the 30-day span after accounting for age, COVID-19 severity, NLR, and comorbidity (*p* = 0.009).

Univariate Cox regression analysis showed that endothelin-1 lost its prognostic significance in the analysis of long-term mortality (*p* = 0.304) (Table 3). In the multivariate analysis, the significance of such confounders as NLR and age remained.

**Figure 2:**
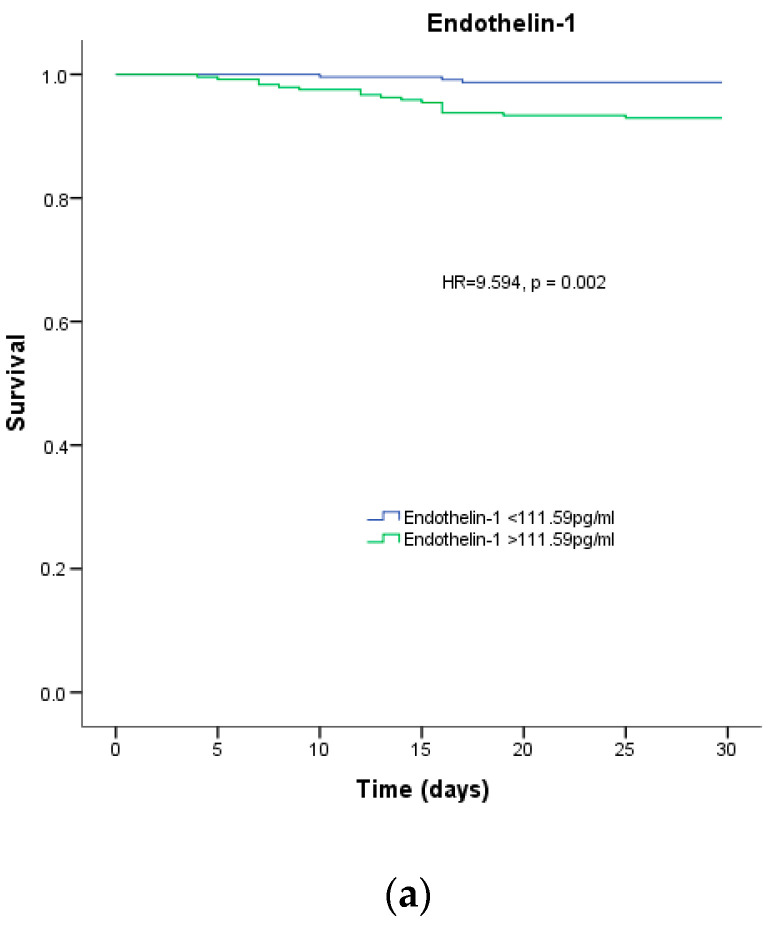

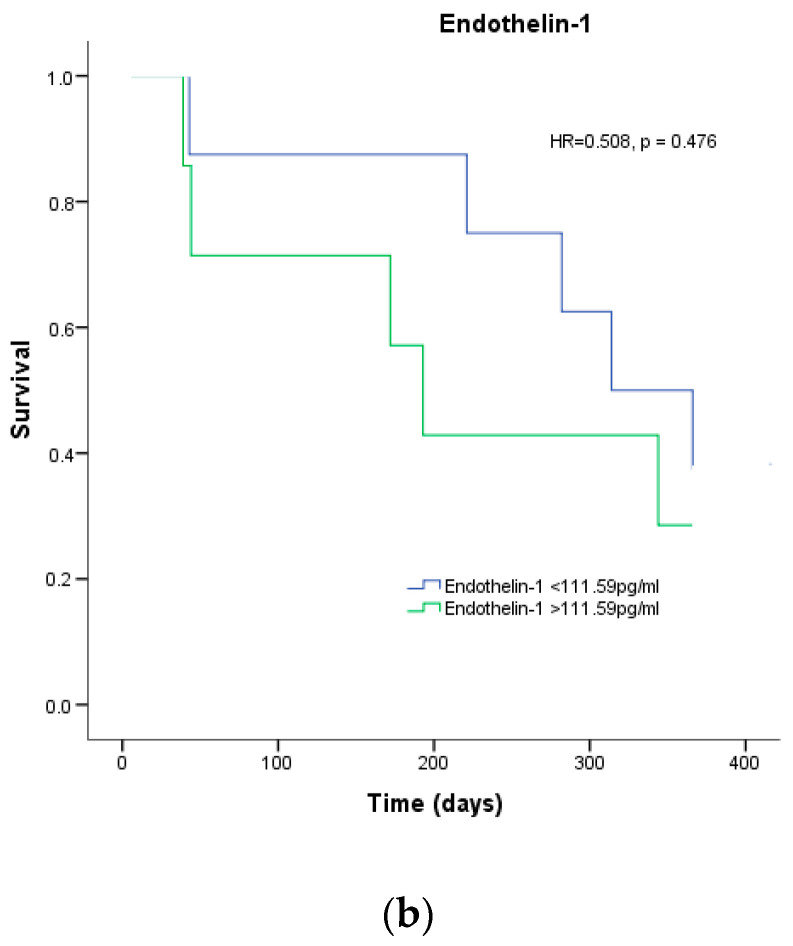
Survival analysis. Percentage of survival of patients with COVID-19 depending on the median endothelin-1 for (**a**) 1-month mortality and (**b**) 12-month mortality.

According to a survival analysis conducted within the first month, patients with plasma endothelin-1 levels higher than the median concentration of 111.59 pg/mL had a greater risk of in-hospital mortality (HR = 9.594, *p* = 0.002), as shown in Figure 2A. However, the survival analysis for patients who passed away within 12 months did not reveal any statistical significance (*p* = 0.476), as depicted in Figure 2B.

## 4. Discussion

This study hypothesized that increased serum ET-1 levels during the acute phase of COVID-19 are associated with the risk of patient mortality during the acute period and at 12 months of follow-up. 

It was determined that ET-1 levels have prognostic significance in the risk of mortality in hospitalized patients in the acute period of the disease, and this association persisted after adjustment for such indicators as age, severity, NLR, and the presence of comorbidities.

The correlation between higher ET-1 levels and severe COVID-19 illness, leading to increased mortality rates during the acute phase, is backed by this study and previous studies. Willems et al. demonstrated [23] that COVID-19 patients exhibited elevated levels of ET-1 in comparison to the control group. Furthermore, Abraham et al. [22] observed that patients, who were admitted to the hospital, including those who succumbed to the illness or experienced acute myocardial infarction or kidney damage, displayed notably high levels of plasma ET-1 during the acute phase of the disease.

One of the mechanisms for increasing plasma ET-1 levels may be the inflammatory cytokine-induced release of specific endothelial cell granules Weibel–Palade bodies (WPB) which is considered one of the biological mechanisms by which SARS-CoV- 2 causes EC activation and damage. This subcellular organelle was originally defined as the intracellular pool of the von Willebrand factor, and it contains a greater number of endothelial cell markers, including ET-1. Moreover, Druml et al. reported significantly increased plasma ET-1 levels in acute respiratory distress syndrome indicating increased production and decreased degradation of endothelin in acute respiratory distress syndrome (ARDS) [32]. In addition, the early treatment with the endothelin receptor antagonist bosentan within three days after the first symptoms showed effectiveness in preventing severe COVID-19 in patients from the high-risk group [33]. 

Gregoriano et al. previously reported the lack of prognostic value of C-terminal proendothelin-1 (proET-1) for predicting mortality in COVID-19 [27]. ProET-1 does not cause vasoconstriction like ET-1 because it is an inactive peptide metabolized differently. Accordingly, these differences may make comparing the two biomarkers difficult in vivo.

SARS-CoV-2 infection promotes endothelial induction due to direct entry of viral bodies and host inflammatory response. Moreover, in COVID-19 patients, endothelial cell damage may be caused by the induction of apoptosis and pyroptosis. COVID-19 endotheliitis could potentially result in systemic microcirculatory dysfunction in different vascular beds, leading to clinical consequences. A significant increase in antibody levels to angiotensin I and endothelin I receptors is considered a consequence of severe damage to the vascular endothelium in a pro-inflammatory environment [34]. An alternative explanation might be increased antibody levels in high-risk COVID-19 groups with underlying cardiovascular disease, hypertension, and older age. Probably, COVID-19 endotheliitis, when layered on existing endothelial dysfunction in diseases like COPD and diabetes, significantly increases fatal outcomes. Our study confirmed the relationships between the risk of 30-day mortality and such co-founders as age, disease severity, previous myocardial infarction, and an increase in NLR described in the previous studies [35,36,37]. 

We did not find that serum ET-1 levels measured in the acute phase of COVID-19 are closely associated with the risk of mortality at a follow-up period of 12 months. Moreover, the ET-1 level in surviving patients did not differ significantly from the deceased group in the post-COVID period.

Dalla Sega et al. [28] reported that endothelin level was higher in survived patients during the acute phase in comparison with the deceased, which was also unexpected. It was suggested that the increasing level of ET-1 could be explained by rising lung hypertension [38] and the development of the angiogenesis that has been observed in the lungs of COVID-19 patients [39]. Subsequently, authors have assumed that higher levels of ET-1 induce angiogenesis in survived patients, while the clinical impact of angiogenesis in COVID-19 is not established yet [40]. On the other side, ET-1 mediates vasoconstriction by endothelin receptor A (ET_A_), which is located in the smooth muscles of vessels. Notably, endothelial cells decrease the regulation of ACE-1 and ET-1 released during shear stress and high blood flow in vitro [41]. To sum up, we speculate that lower ET-1 levels in the deceased group might be explained by the occurrence of high shear stress due to chronic inflammation or endothelial damage [42]. In addition, it could be indirectly confirmed by the association between the risk of long-term mortality with the NLR index and the age of the patients.

Our study has several limitations including a small deceased group size, which could affect the detection of significant associations. However, the sample size was sufficiently representative to detect significant associations between age, NLR, and incident death at 12 months of follow-up.

Furthermore, patients in the survived and deceased groups within 12 months had a comparable incidence of chronic diseases affecting ET-1 levels (hypertension, diabetes mellitus, coronary heart disease, heart failure, chronic kidney disease), and their basal ET-1 levels were unknown. Henkens M. et al. reported that pre-existing comorbidities, commonly found in older people, make a minimal contribution to in-hospital mortality compared to age. Moreover, the contribution of comorbidities to outcomes in <60-year-old patients was restricted only to cardiovascular disease and had no significant association with diabetes, CKD, and COPD [43].

Also, the study was limited by the absence of an analysis on how increased mortality from various causes is associated with ET-1 levels. The analysis of the causes of death among 3704 patients showed that patients who had recovered from SARS-CoV-2 were at an increased risk of respiratory diseases (aHR 1.9, 95% CI 1.2–3.0), malignancies (aHR 1.5, 95% CI over 12 months 1.2–1.9), and death from cardiovascular diseases (aHR 2.1, 95% CI 1.8–2.3) [6]. In this study, the most common causes of death in the post-COVID period were cardiovascular failure (46.6%), acute vascular events (33.3%), and malignant neoplasms (17.65%). The small sample size in each group was the most limiting point for conducting an in-depth analysis of ET-1 levels depending on the causes of mortality. Considering multiorgan damage after SARS-CoV-2 determination, death from any cause within a year merged with the deceased group.

## 5. Conclusions

In summary, this study confirms that ET-1 levels are associated with increased mortality risk during the acute period of COVID-19. This may provide a basis for further research into targeted therapy with endothelin antagonists. It is suggested that plasma ET-1 level association with COVID-19 survivor mortality risk does not persist after 12 months of follow-up. However, it remains unclear which markers of endothelial dysfunction are dysregulated in COVID-19 survivors and can be used as biomarkers to diagnose and prevent the long-term impact of SARS-CoV-2 infection.

## Figures and Tables

**Table 1:** Patient characteristic validation cohort.

Baseline Variable	Survivors, *n* = 435	Non-Survivors 1-Month, *n* = 20	Non-Survivors 12-Months, *n* = 15	*p*-Value *	*p*-Value **
	Ме (Q25–Q75)	Ме (Q25–Q75)	Ме (Q25–Q75)		
Demographic					
Age (y)	61 (50–69)	73 (65–81)	72 (62–84)	0.0001	0.002
Gender, *n* (%)					
Male	163 (37.5)	11 (55)	5 (33.3)	0.112	0.745
Female	272 (62.5)	9 (45)	10 (66.7)		
Comorbidities or coexisting disorders, *n* (%)					
Hypertension	221 (50.8)	15 (75)	7 (46.7)	0.033	0.753
Chronic heart failure	149 (34.3)	14 (70)	5 (33.3)	0.001	0.941
Diabetes mellitus	74 (17.0)	6 (30)	4 (26.7)	0.148	0.332
Chronic obstructive pulmonary disease	11 (2.5)	1 (5)	0	0.244	0.621
Myocardial infarction in medical history	10 (2.3)	5 (25)	6 (17.1)	0.0001	0.282
Chronic renal failure	15 (3.4)	2 (10)	0	0.119	0.465
Malignancy	13 (3.0)	0	2 (13.3)	0.407	0.023
BMI (kg/m^2^)	28.3 (24.7–32.4)	26.5 (23.9–32.4)	29.0 (23.1–34.5)	0.531	0.649
Vital signs at day of sampling					
Heart rate (bpm)	80 (76–86)	82 (76–91)	78 (76–88)	0.171	0.837
Respiratory rate (vpm)	19 (18–20)	22 (20–23)	20 (19–22)	0.0001	0.015
Oxygen saturation, %	96 (94–98)	93 (91–98)	95 (92–98)	0.033	0.437
Severity, *n* (%)					
Moderate	364 (83.7)	7 (35)	11 (73.3)	0.0001	0.291
Severe	71 (16.3)	13 (65)	4 (26.7)		
Invasive mechanical ventilation, *n* (%)	9 (2.1)	12 (60)	0	0.0001	0.574
ICU admission (*n*, %)	40 (9.2)	13 (65.0)	2 (13.3)	0.0001	0.588
Infiltrate on chest radiograph, %	25 (12–40)	45 (25–55)	25 (15–45)	0.005	0.609
Hospital length of stay (days)	10 (8–11)	11 (7–14)	10 (9–15)	0.230	0.169
Laboratory findings					
Hemoglobin (g/L)	139 (129–153)	134 (125–156)	143 (118–153)	0.452	0.729
Leukocytes × 10^9^/L	4.9 (3.8–6.2)	5.9 (4.2–8.2)	6.1 (5.1–7.0)	0.144	0.01
Neutrophils × 10^9^/L	3.08 (2.19–4.25)	4.36 (2.83–6.17)	4.02 (3.54–5.51)	0.025	0.005
NLR	2.4 (1.6–3.5)	3.8 (2.5–8.7)	3.0 (2.0–9.1)	0.001	0.09
Platelets × 10^9^/L	187 (155–218)	189 (155–216)	196 (176–210)	0.656	0.481
ESR, (mm/h)	15 (10–23)	15 (10–22)	14 (4–20)	0.836	0.243
Creatinine (mmol/L)	88 (80–96)	89 (77–99)	86 (82–97)	0.780	0.967
ALT (units/L)	29 (23–33)	24 (19–31)	26 (18–32)	0.100	0.169
AST (units/L)	26 (23–35)	27 (25–32)	30 (24–34)	0.871	0.378
Bilirubin (µmol/L)	13.0 (11.8–15.0)	14.0 (12.0–16.0)	14.9 (11.6–16.0)	0.462	0.341
Glucose (mmol/L)	6.5 (5.5–7.9)	6.2 (5.1–12.2)	8.3 (6.5–16.5)	0.929	0.024
CRP (mg/l)	12 (6–52)	39.9 (9.1–139.2)	24 (6–103)	0.062	0.596
Ferritin (μg/l)	218 (130–364)	244 (206–681)	242 (54–395)	0.077	0.515
D-dimer, (ng/mL)	281 (165–424)	434 (319–538)	379 (231–582)	0.002	0.05
Endothelin-1, (pg/mL)	100.4 (27.7–197.8)	188.4 (130.6–263.6)	84.7 (37.6–163.6)	0.001	0.643
Treatment					
Anticoagulants	392 (89.9)	18 (90)	14 (93.3)	0.997	0.664
Glucocorticosteroids	323 (53.2)	17 (85)	10 (66.7)	0.006	0.305
Antibiotic therapy	308 (70.6)	18 (90)	14 (93.3)	0.070	0.056
Antiviral therapy	93 (2)	8 (40)	3 (20)	0.050	0.895

Notes: * Survivors versus 1-month non-survivors COVID-19 patients. ** Survivors versus 12-month non-survivors COVID-19 patients. Abbreviations: BMI, body mass index; ICU, intensive care unit; CRP, C-reactive protein; ESR, erythrocyte sedimentation rate; ALT, alanine transaminase; AST, aspartate transaminase.

**Table 2:** Results of univariate and multivariate Cox regression analysis to assess risk factors for 1-month mortality in patients with COVID-19.

Marker	Univariate Analysis			Multivariate Analysis		
	HR	CI	*p*-Value	HR	CI	*p*-Value
Endothelin-1	1.002	1.000–1.004	0.036	1.004	1.001–1.007	0.009
Severity	8.538	3.406–21.403	0.0001	5.765	2.242–14.823	0.0001
Gender (Male)	2.032	0.842–4.903	0.115	-	-	-
NLR	1.118	1.065–1.173	0.0001	1.093	1.031–1.158	0.003
Age	1.073	1.035–1.112	0.0001	1.076	1.029–1.126	0.001
Arterial hypertension	2.843	1.033–7.824	0.043	0.745	0.245–2.262	0.603
Myocardial infarction	10.714	3.891–29.505	0.0001	5.742	1.867–17.659	0.002
Chronic heart failure	4.351	1.672–11.323	0.003	2.016	0.425–9.559	0.377
Chronic obstructive pulmonary disease	3.207	0.429–23.960	0.256	-	-	-
Chronic kidney disease	2.958	0.686–12.748	0.146	-	-	-

**Table 3:** Results of univariate and multivariate Cox regression analysis to assess risk factors for 12-month mortality in patients with COVID-19.

Marker	Univariate Analysis			Multivariate Analysis		
	HR	CI	*p*-Value	HR	CI	*p*-Value
Endothelin-1	0.996	0.990–1.003	0.304	-	-	-
Severity	3.290	0.787–13.757	0.103	-	-	-
Gender (Male)	1.126	0.318–3.989	0.854	-	-	-
NLR	1.106	1.019–1.200	0.016	1.093	1.003–1.192	0.043
Age	1.069	1.017–1.124	0.009	1.070	1.015–1.128	0.012
Arterial hypertension	0.646	0.182–2.288	0.498	-	-	-
Myocardial infarction	4.431	0.561–34.973	0.158	-	-	-
Chronic heart failure	0.819	0.212–3.165	0.772	-	-	-

## Data Availability

Deidentified information data presented in this manuscript will be made available 6 months after publication on reasonable request by email to the corresponding author for research purposes.
